# Longitudinal assessment of cardiac parameters through MRI in breast cancer patients treated with anti-HER2 therapy

**DOI:** 10.1186/s41747-023-00338-9

**Published:** 2023-05-15

**Authors:** Sainan Cheng, Jianwei Wang, Yawen Wang, Linlin Qi, Fenglan Li, Jianing Liu, Jiaqi Chen, Yang Fan, Lizhi Xie

**Affiliations:** 1 Department of Diagnostic Radiology, National Cancer Center / Cancer Hospital, Chinese Academy of Medical Sciences and Peking Union Medical College, #17 Panjiayuan Nanli, Chaoyang District, Beijing, 100021 China; 2 MR Research China, GE Healthcare, Beijing, China

**Keywords:** Breast neoplasms, Cardiotoxicity, Magnetic resonance imaging, Trastuzumab, Pertuzumab

## Abstract

**Background:**

We evaluated the early changes in left ventricular (LV) volumetric, functional, and tissue characteristics in human epidermal growth factor receptor 2 (HER2)-positive breast cancer patients treated with trastuzumab and/or pertuzumab at cardiac magnetic resonance imaging (MRI).

**Methods:**

HER2-positive breast cancer patients undergoing planned anti-HER2 therapy and nonanthracycline-based chemotherapy were enrolled and subdivided into dual anti-HER2 (trastuzumab plus pertuzumab) group and trastuzumab group. Cardiac MRI was performed before treatment and three months after starting, covering ventricular volumes, cardiac function, systolic myocardial strain, myocardial oedema, and T1 and T2 relaxation times. Cardiac dysfunction was primarily defined as a > 10% reduction in LV ejection fraction (LVEF) to < 55% and/or a > 15% global longitudinal strain (GLS) change at the follow-up MRI examination.

**Results:**

Twenty-four HER2-positive patients were evaluated (16 in the dual anti-HER2 group, 8 in the trastuzumab group). Six patients developed cardiac dysfunction at follow-up, five of them in the dual anti-HER2 group. One patient developed symptomatic heart failure, and five patients developed asymptomatic cardiac dysfunction. Patients displayed significantly decreased systolic function and increased T1 and T2 relaxation time at follow-up (*p* ≤ 0.031). Systolic dysfunction remained significant in the dual anti-HER2 group. The decrease in GLS in the trastuzumab group was not significant (*p* = 0.169). T1 and T2 relaxation times tended to increase, but this was not significant at subgroup analysis.

**Conclusions:**

Cardiac MRI scans showed frequent signs of subclinical cardiotoxicity after short-term anti-HER2 therapy and nonanthracycline-based chemotherapy; the effect was slightly stronger in patients treated with dual therapy.

**Key points:**

• A frequent subclinical cardiotoxicity was detected by cardiac magnetic resonance imaging after short-term anti-human epidermal growth factor receptor 2 (HER2) therapy.

• The change in myocardial strain was more marked in patients treated with dual (trastuzumab plus pertuzumab) than with trastuzumab only anti-HER2 therapy.

• Cardiotoxicity surveillance through MRI is an interesting option particularly in patients treated with dual anti-HER2 therapy.

## Background

Approximately 20% of breast cancers (BCs) strongly overexpress human epidermal growth factor receptor 2 (HER2), whose overexpression has been associated with more aggressive disease and a higher risk of recurrence. Trastuzumab, an anti-HER2 extracellular domain IV binding antibody, significantly improves long-term prognosis in HER2-positive BC patients; pertuzumab, an antibody that binds to HER2 extracellular domain II, has demonstrated promising efficacy in both metastatic and early BC and is an integral part of HER2-positive BC therapy [1, 2]. Despite the impressive anticancer benefit, the cardiotoxicity of trastuzumab and pertuzumab frequently limits their clinical use and may reduce the quality of life of BC survivors [3]. Early recognition of subclinical cardiotoxicity allows clinicians to incorporate cardioprotective therapy before there is a significant decline in cardiac systolic function, which may or may not be reversible, and decreases the risk of interruptions in cancer therapy, which could otherwise affect patient survival [4].

Cardiac magnetic resonance imaging (MRI) is the imaging modality of choice in this setting, particularly due to its capabilities of accurate cardiac function measurement and advanced tissue characterisation. Therefore, many studies have aimed to explore the effect of anti-HER2 therapy on cardiac function using cardiac MRI. An early ≥ 10% deterioration in global longitudinal strain (GLS) has been associated with a lower left ventricular ejection fraction (LVEF) at 9 months [5]. In one study, earlier relative changes in cardiac MRI strain and LVEF were prognostic for subsequent cancer therapy-related cardiovascular dysfunction (CTRCD) [6], suggesting that the first 3 months of trastuzumab therapy are the most critical period for the development of cardiotoxicity.

However, most prior studies used only one anti-HER2 target therapy (trastuzumab), and a large part of the participants had been previously treated with anthracyclines. Increasing use of trastuzumab plus pertuzumab has led to further evaluation of anti-HER2 therapy-related cardiotoxicity without being affected by anthracycline chemotherapy, which may also cause myocardial injury [7]. In addition, data on early changes in left ventricular (LV) volumetric, functional, and tissue characteristics in patients treated with anti-HER2 target therapy and nonanthracycline-based chemotherapy are limited.

In this prospective study, HER2-positive BC participants treated with trastuzumab and/or pertuzumab in combination with nonanthracycline-based chemotherapy underwent cardiac MRI immediately before treatment initiation as well as 3 months after the start of treatment. The purpose was to evaluate and compare the early changes in LV volumetric, functional, and tissue characteristics in patients following short-term exposure to trastuzumab and/or pertuzumab using cardiac MRI.

### Methods

## Study participants

Between January 2021 and May 2022, women (aged 18 years and older) with HER2-positive BC scheduled to receive anti-HER2 therapy were prospectively recruited at our hospital. Anticancer treatments were managed at the discretion of the oncologist. The chemotherapy and anti-HER2 regimen consisted of taxane plus carboplatin plus trastuzumab plus pertuzumab or taxane plus carboplatin plus trastuzumab every 3 weeks. The exclusion criteria were anthracycline-based chemotherapy, previous treatment with anticancer therapy, preexisting cardiac disease, contraindications to cardiac MRI and refusal to participate in the study.

Patients were subdivided into two groups according to the anti-HER2 regimen: dual anti-HER2 group (trastuzumab plus pertuzumab) and trastuzumab alone group. The demographic characteristics, cardiovascular risk factors, adjuvant treatment regimens, and BC characteristics were collected at baseline in a standardised manner. Cardiac MRI examinations were performed at baseline and 3 months after initiation of anti-HER2 therapy. Only participants who completed the entire study protocol were included in the final analysis. The hospital research ethics committee approved this study (National Cancer Center, Beijing, China, No. NCC2020C-508), and all study participants provided written informed consent.

The presence of CTRCD was primarily defined as a > 10% reduction in LVEF to < 55% or a > 15% GLS change at the follow-up MRI examination [8–10]. A relative reduction in GLS of 15% from baseline was considered abnormal and a marker of early LV subclinical dysfunction [11].

### Cardiac MRI protocol

Cardiac MRI examinations were performed with a clinical 3-T scanner (SIGNA Architect, GE Healthcare). A 30-channel coil was used for signal reception. All images were acquired in a breath-hold by using a three-lead vector cardiogram for cardiac synchronisation. Cine steady-state free-precession cine images (repetition time 3.7 ms; echo time 1.7 ms; 16 views per segment; flip angle 60 degrees) were obtained in the short axis, two-chamber, three-chamber and four-chamber views for functional analysis. T2-weighted dual inversion-recovery imaging (repetition time equal to 2–3 heartbeats; echo time 70 ms) was used for visualisation of myocardial oedema. A modified look-locker inversion-recovery approach was used for T1 mapping; images were acquired with a 3(3)3(3)5 heartbeat pattern (echo time 1.3 ms; repetition time 2.9 ms; flip angle 35 degrees; first inversion time 140 ms; inversion time increment 80 ms). T2 mapping was conducted using a double inversion-recovery multi-echo fast spin-echo sequence with four different echo times (10.8, 32.4, 54.0, and 75.5 ms; echo train length 16; flip angle 90 degrees; bandwidth 83.33 kHz).

### Image analysis

All images were transferred to the commercially available software cvi42 (Circle Cardiovascular Imaging Inc.) for blinded analysis. The epicardial and endocardial borders of the LV myocardium were manually traced in all phases on short-axis cine images to calculate functional parameters (LVEF, LV end-diastolic volume index, and LV end-systolic volume index, cardiac index, stroke volume index, LV mass index). Papillary muscles were included in the volume. T1 and T2 values were measured using a region of interest placed in the septum of the mid short-axis slice. For feature-tracking strain analysis, endocardial and epicardial contours were manually traced from cine images in the end-diastolic phase. Three long-axis cine images (two-chamber, three-chamber and four-chamber cine images were tracked to derive GLS, while short-axis cine images were used to derive global radial strain (GRS) and global circumferential strain (GCS). All images were analysed by one radiologist with 7 years of experience in cardiac MRI who was blinded to all the clinical information. To assess reproducibility, LVEF, GRS, GCS, GLS, T1 and T2 relaxation time analyses were performed again in 12 randomly selected patients by a second observer (with 9 years of experience) and the first radiologist after an interval of at least 1 month.

### Statistical analysis

Clinical and cardiac MRI characteristics were checked for normal distribution using the Shapiro‒Wilk test. Continuous data are shown as mean ± standard deviation for normally distributed values and medians with interquartile ranges (IQRs) for nonnormally distributed variables. Categorical variables are reported as numbers with percentages. For intraindividual comparison of variables between baseline and follow-up MRI examinations, the paired test or Wilcoxon signed-rank test was used, as appropriate. Analysis was carried out in each group separately as well as in the entire study group. For comparison of variables between groups, the unpaired *t* test or Mann‒Whitney *U* test was used, as appropriate. Bland–Altman plots and intraclass correlation coefficients with 95% confidence intervals were used to assess inter- and intra-observer variabilities. Statistical analyses were performed with SPSS (version 26, IBM, Chicago, Illinois, USA) and GraphPad Prism (version 9.3.0, GraphPad Software, San Diego, CA, USA); *p*-values lower than 0.05 were considered indicative of significant difference.

## Results

### Participant characteristics

**Fig. 1:**
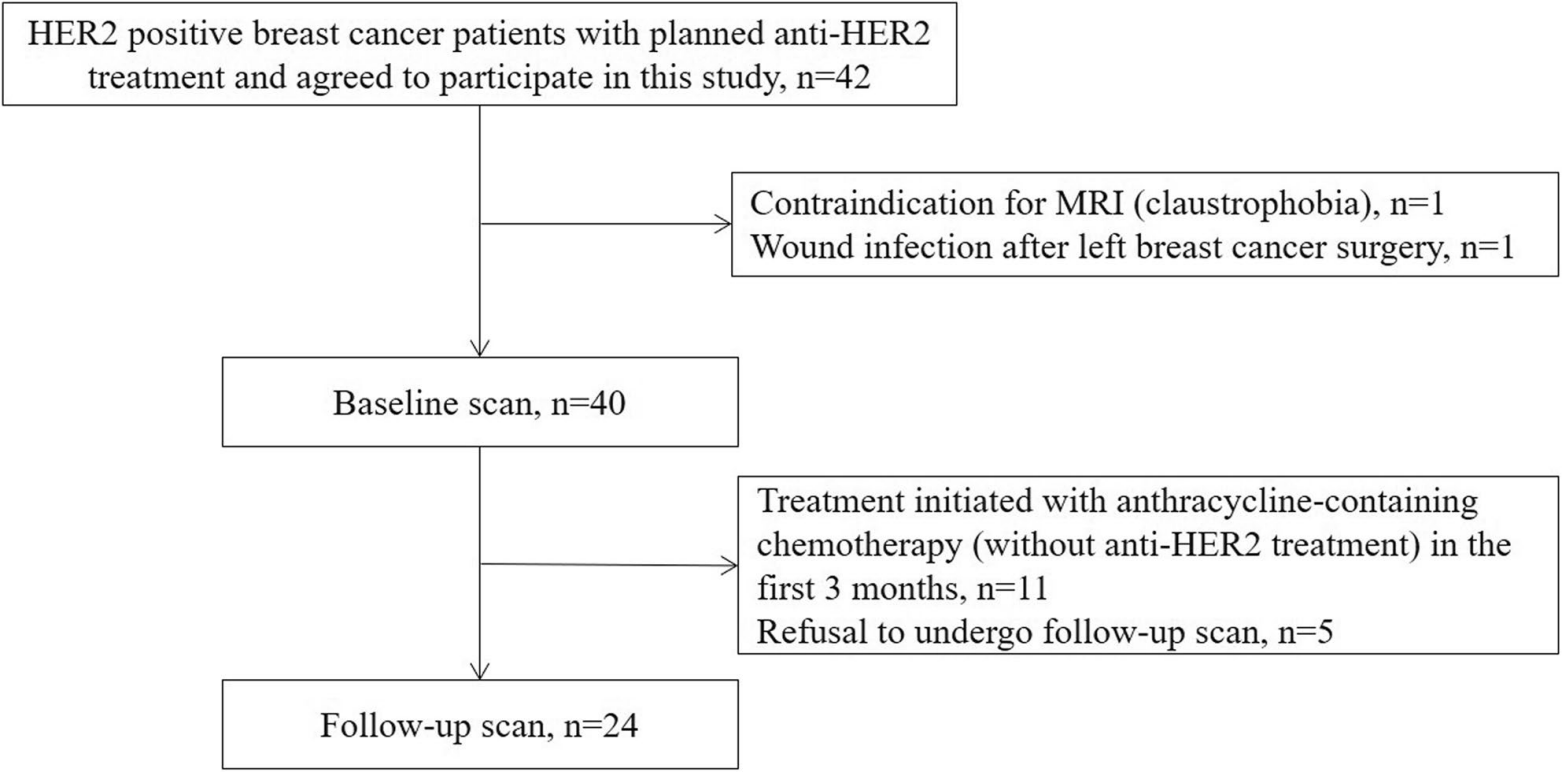
Study flow diagram

**Fig. 2:**
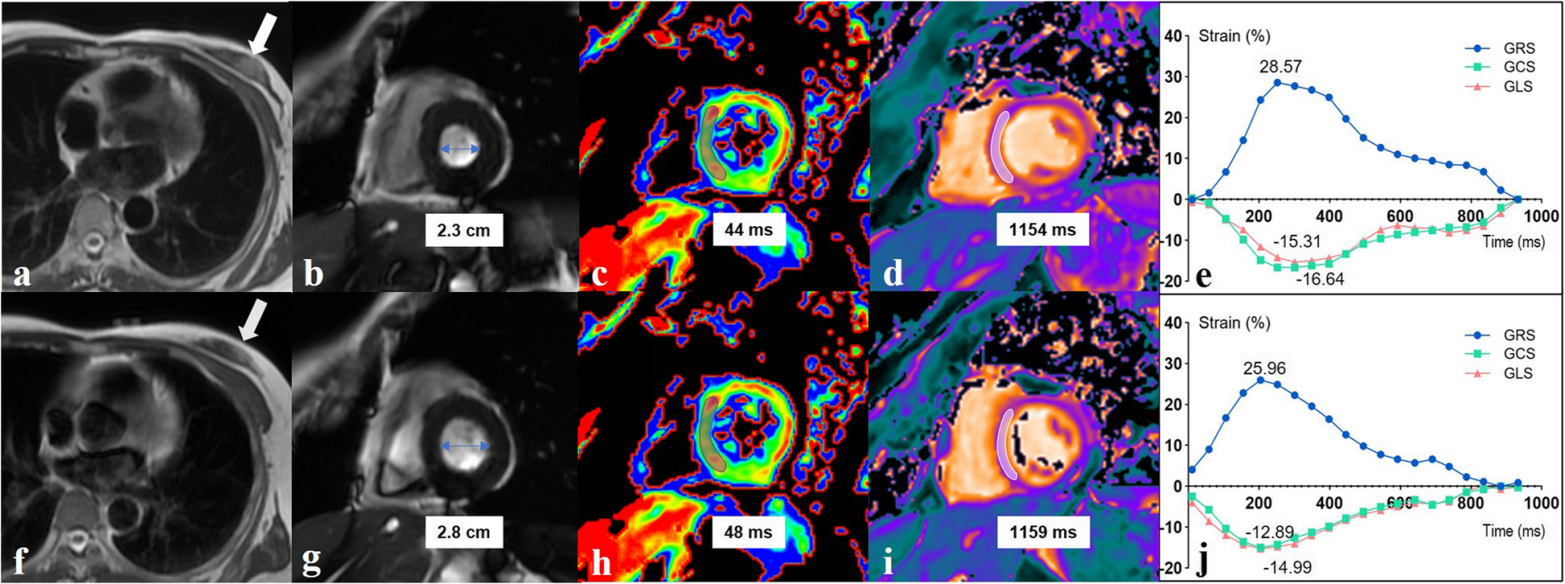
Cardiac magnetic resonance imaging in a 59-year-old woman with human epidermal growth factor receptor 2 positive left-side breast cancer. Left ventricular ejection fraction was 66% at baseline and 56% at follow-up. Top images (**a**–**e**) are at baseline. Bottom images (**f**–**j**) are after four cycles of the taxanes plus carboplatin plus trastuzumab plus pertuzumab regimen (95 days after the baseline scan). Axial T2-weighted images (**a**, **f**) show a decrease in the tumour size of the left breast (arrows). Short-axis cine images (**b**, **g**) demonstrate an increase in left ventricular end-systolic internal diameter (2.3 cm at baseline, 48 cm at follow-up). T2 mapping images (**c**, **h**) show a slight increase in T2 relaxation time in the septum of the mid short-axis slice (44 ms at baseline, 48 ms at follow-up). T1 mapping images (**d**, **i**) show a slight increase in T1 relaxation time in the septum of the mid short-axis slice (1,154 ms at baseline, 1,195 ms at follow-up). Global radial, circumferential and longitudinal strains before and after four cycles of therapy (**e**, **j**). *GCS* Global circumferential strain, *GLS* Global longitudinal strain, *GRS* Global radial strain

A total of 24 patients were included (mean age ± standard deviation, 47 years ± 11) (Fig. 1). Of the 24 HER2-positive BC patients, 16 (67%) received taxanes plus carboplatin plus trastuzumab plus pertuzumab and 8 (33%) received taxanes plus carboplatin plus trastuzumab treatment. The mean time ± standard deviation between the baseline and follow-up cardiac MRI scans was 107.5 ± 19.7 days. The baseline characteristics are shown in Table 1. A clinical example is shown in Fig. 2.

**Table 1:** Clinical characteristics and treatment of the study participants

*Variable*	*Value*
No. of participants	24
Demographics ^a^	
Age (years)	47.2 ± 11.1
Height (cm)	161.0 ± 4.3
Weight (kg)	61.9 ± 9.1
Body surface area (m^2^)	1.62 ± 0.13
Cardiovascular risk factors ^b^	
Obesity (body mass index > 30 kg/m^2^)	3 (12.5)
Age 65–79 years	2 (8.3)
Arterial hypertension	1 (5.0)
Diabetes	0 (0.0)
Prior cardiovascular disease	0 (0.0)
HFA-ICOS baseline cardiovascular toxicity risk stratification ^b^	
Moderate risk (with a total of 2 points)	2 (8.3)
Low risk	22 (91.7)
No risk factors	19 (79.2)
One moderate risk with a total of 1 point	3 (12.5)
Cardiac medication	0 (0.0)
Left-/right-sided breast cancer ^b^	15 (62.5)/9 (37.5)
Anti-HER2 therapy ^b^	
Trastuzumab	8 (33.3)
Trastuzumab and pertuzumab	16 (66.7)
Interval time between baseline and follow-up MRI (days)	107.5 ± 19.7

^a^ Data are given as mean ± standard deviation. ^b^ Data in parentheses are percentages. *HER2* Human epidermal growth factor receptor 2; *HFA-ICOS* Heart Failure Association-International Cardio-Oncology Society, *MRI* Magnetic resonance imaging

### Clinical manifestations and CTRCD

One patient had arterial hypertension at baseline, but the blood pressure was well controlled by appropriate medication. No participant had a history of diabetes or prior cardiovascular disease. No patient showed fatigue, dyspnoea or orthopnoea at baseline. Baseline cardiovascular toxicity risk assessments were conducted according to the Heart Failure Association–International Cardio-Oncology Society tools [4]. Two patients showed moderate risk (with a total of 2 points) and 22 patients showed low risk.

Of 24 patients, 6 (25%) had developed CTRCD at the follow-up MRI examination (2 patients showed both LVEF decline > 10% to less than 55% and GLS change > 15%, 4 patients showed GLS change > 15%). One patient developed symptomatic heart failure (LVEF 35%) during follow-up. The main symptoms were fatigue and dyspnoea. Five patients experienced an asymptomatic decrease in LVEF. Five of the six patients were on dual (trastuzumab and pertuzumab) anti-HER2 therapy, and one patient was on single (trastuzumab) anti-HER2 therapy (Table 2).

**Table 2:** Cancer therapy–related cardiac dysfunction of the entire study group and subgroups

Variable	Entire study group (*n* = 24)	Dual anti-HER2 group (*n* = 16)	Trastuzumab group (*n* = 8)
LVEF decline > 10% to less than 55%	2 (8.33)	2 (8.33)	0 (0.0)
GLS change > 15%	6 (25.00)	5 (20.83)	1 (4.17)
Symptomatic heart failure	1 (4.17)	1 (4.17)	0 (0.0)

Data in parentheses are percentages out of the entire group. *GLS* Global longitudinal strain; *HER2* Human epidermal growth factor receptor 2, *LVEF* Left ventricular ejection fraction

### Volumetric, functional, and tissue characteristics between baseline and follow-up

The median LVEF was significantly lower at follow-up than at baseline (63.0%, IQR 61.3–66.8%) *versus* 59.0% (IQR 55.0–60.0%) (*p* < 0.001). The LV end-systolic volume index was higher at follow-up than at baseline MRI (median 22.1 mL/m^2^, IQR, 18.8–28.6 mL/m^2^) *versus* 25.6 mL/m^2^ (IQR 21.6–31.9 mL/m^2^) (*p* = 0.001). The stroke volume index (42.3 mL/m^2^ ± 7.0 at baseline *versus* 36.8 mL/m^2^ ± 5.8 at follow-up, *p* < 0.001) and cardiac index (3.20 mL/m^2^ ± 0.61 at baseline *versus* 2.86 mL/m^2^ ± 0.50 at follow-up, *p* = 0.003) were lower at the follow-up than at the baseline MRI. We found no evident differences between baseline and follow-up MRI examinations in the LV end-diastolic volume index (*p* = 0.880) or LV mass index (*p* = 0.536).

At myocardial strain analysis, lower GRS (33.2% ± 7.1% *versus* 29.3% ± 6.0%, *p* < 0.001), GCS (-18.8% ± 2.4 *versus* -17.4% ± 2.4%, *p* < 0.001) and GLS (-16.0% ± 1.6 *versus* -15.0% ± 2.0, *p* = 0.004) were observed at follow-up.

No obvious myocardial oedema was detected on T2-weighted short-tau inversion-recovery images.

**Fig. 3:**
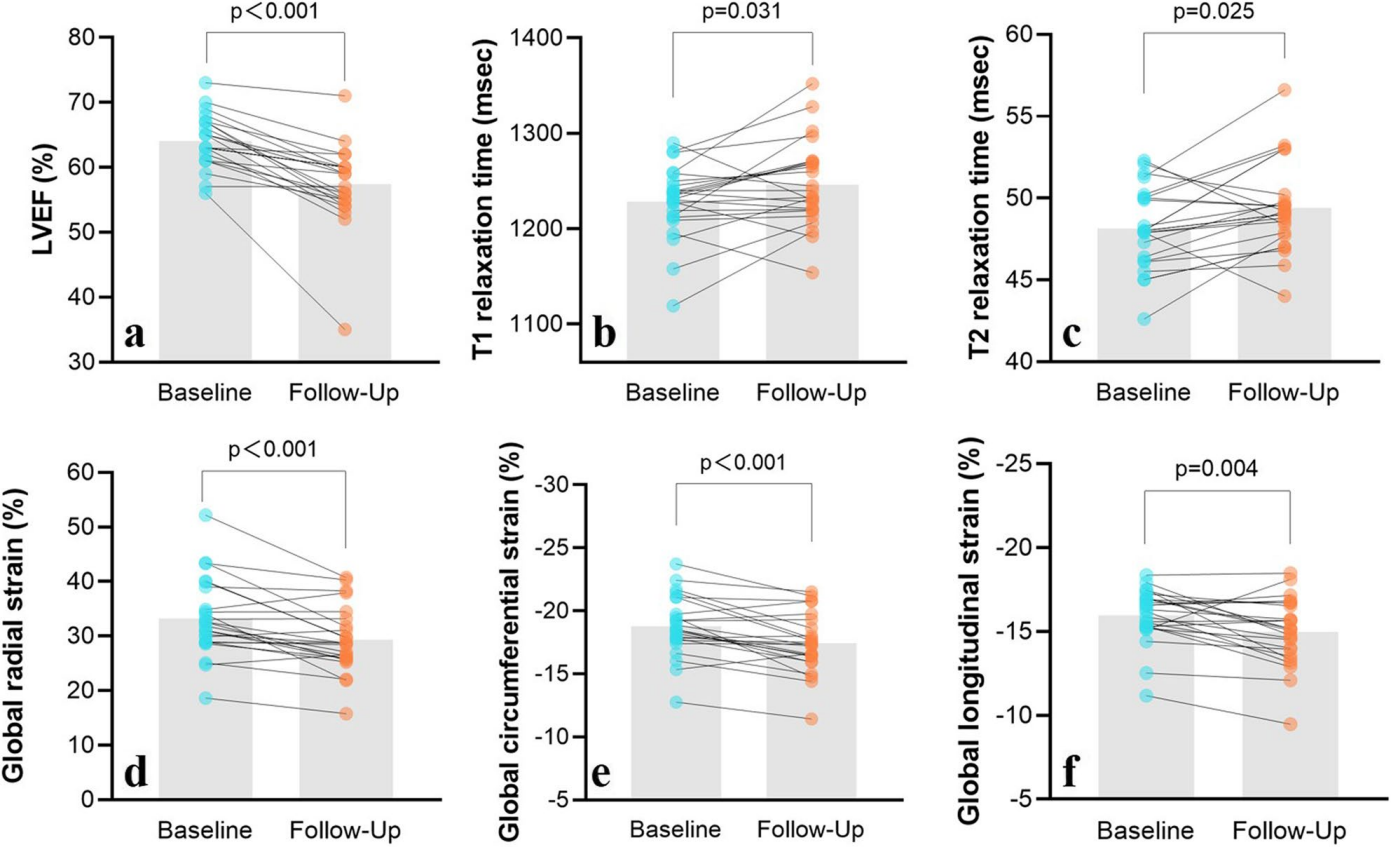
Bar graphs of cardiac magnetic resonance imaging parameters at baseline and follow-up. Bars represent median data in the first graph and represent mean data in other graphs. Blue plots represent intraindividual values at baseline, and orange plots represent intraindividual values at the follow-up. *LVEF* Left ventricular ejection fraction

We found a significant increase in T1 relaxation time (1,228.4 ms ± 37.8 *versus* 1,246.34 ms ± 44.7, *p* = 0.031) and T2 relaxation time (48.1 ms ± 2.5 *versus* 49.4 ms ± 2.8, *p* = 0.025) between the baseline and follow-up MRI examinations (Fig. 3).

At subgroup analysis, lower LVEF, stroke volume index and strain rates (GRS, GCS) were observed in each subgroup. The cardiac index and GLS decreased significantly at follow-up in the dual anti-HER2 group but not in the trastuzumab group. T1 and T2 relaxation times showed a tendency towards an increase, but this was not statistically significant. There was no significant change in the LV end-diastolic volume index or LV mass index in either group at follow-up (Table 3).

**Table 3:** Cardiac magnetic resonance imaging results at baseline and follow-up

Variable	Anti-HER2 treatment (*n* = 24)			Trastuzumab and pertuzumab treatment (*n* = 16)			Trastuzumab treatment (*n* = 8)		
	Baseline	Follow-up	*p*-value	Baseline	Follow-up	*p*-value	Baseline	Follow-up	*p*-value
LV ejection fraction (%)	63.00 (61.3, 66.8)	59.0 (55.0, 60.0)	< 0.001	63.0 (61.0, 65.8)	57.5 (55.0, 60.0)	< 0.001	65.3 ± 4.0	58.5 ± 3.8	0.002
LV end-diastolic volume index (mL/m^2^)	64.7 ± 17.3	65.1 ± 13.3	0.880	68.6 (53.5, 73.9)	62.4 (53.5, 74.2)	0.836	68.8 ± 13.5	65.9 ± 10.6	0.253
LV end-systolic volume index (mL/m^2^)	22.1 (18.8, 28.6)	25.6 (21.6, 31.9)	0.001	23.8 (18.9, 28.6)	26.76 (21.2, 31.9)	0.015	23.7 ± 6.0	27.6 ± 6.5	0.005
LV mass index (g/m^2^)	39.4 ± 9.3	38.9 ± 8.5	0.536	40.2 ± 10.5	39.5 ± 9.4	0.544	37.8 ± 6.6	37.5 ± 6.5	0.854
Stroke volume index (mL/m^2^)	42.3 ± 7.0	36.8 ± 5.8	< 0.001	40.9 ± 6.4	36.01 ± 6.2	0.006	45.1 ± 7.7	38.2 ± 4.8	0.007
Cardiac index (L/m^2^)	3.2 ± 0.6	2.9 ± 0.5	0.003	3.0 ± 0.6	2.8 ± 0.5	0.026	3.5 ± 0.6	3.1 ± 0.4	0.075
Global radial strain	33.2 ± 7.1	29.3 ± 6.0	< 0.001	33.1 ± 8.0	28.3 ± 6.1	0.002	33.5 ± 5.2	31.4 ± 5.6	0.045
Global circumferential strain	-18.8 ± 2.4	-17.4 ± 2.4	< 0.001	-18.7 ± 2.7	-17.0 ± 2.5	0.001	-19.1 ± 1.8	-18.3 ± 2.1	0.078
Global longitudinal strain	-16.0 ± 1.6	-15.0 ± 2.0	0.004	-15.7 ± 1.8	-14.5 ± 2.0	0.014	-16.5 ± 0.9	-15.9 ± 1.8	0.169
T1 relaxation time (ms)	1,228.4 ± 37.8	1,246.4 ± 44.7	0.031	1,224.9 ± 44.1	1,241.7 ± 48.2	0.120	1,235.5 ± 20.6	1,255.7 ± 38.0	0.150
T2 relaxation time (ms)	48.1 ± 2.5	49.4 ± 2.8	0.025	48.23 ± 2.25	49.04 ± 2.77	0.191	47.96 ± 2.97	50.2 ± 2.7	0.074

Data are given as mean ± standard deviation or median with interquartile range in parentheses. *HER2* Human epidermal growth factor receptor 2, *LV* Left ventricular

We found no evidence of a difference in any of the MRI parameters between the dual anti-HER2 group and the trastuzumab group at either baseline or follow-up.

### Intra- and interobserver reproducibility

The interobserver correlation coefficient values were 0.90 for LVEF, 0.86 for GRS, 0.84 for GCS, 0.82 for GLS, 0.94 for T1 and 0.86 for T2. The intraobserver correlation coefficient values were 0.93 for LVEF, 0.92 for GRS, 0.86 for GCS, 0.83 for GLS, 0.96 for T1 and 0.90 for T2.

## Discussion

In this preliminary prospective study, we focused on a cohort of BC patients treated with anti-HER2 targeted therapy and nonanthracycline-based chemotherapy. Our results support the evidence of cardiotoxicity as an early treatment effect of anti-HER2 therapy.

Women with HER2-positive BC treated with anti-HER2 therapy are at high risk of cardiac dysfunction, which is considered the main anti-HER2 therapy-related cardiotoxicity in BC patients. Its overall incidence is highly variable, ranging from 7.6% to 28% [12, 13]. Differences in its incidence might be caused by differences in treatment regimens (such as the associated use of anthracyclines, radiotherapy and dual-anti-HEr2 targeted therapy), the definition of cardiotoxicity or CTRCD, and follow-up period. In our study, 8.3% (2/24) of patients developed CTRCD as defined by cardiac MRI-derived LVEF, and 25% (6/24) of patients developed CTRCD as defined by cardiac MRI-derived GLS, which are lower than rates (27% and 42%, respectively) reported in a recent study conducted by Esmaeilzadeh et al. [14], despite the similar definition of CTRCD. In their study, all patients received sequential anthracycline and trastuzumab, and 88% received radiation. Anthracycline and radiation might be associated with a higher rate of LV dysfunction [7]. Conversely in our study, patients received anti-HER2 therapy without anthracycline or left breast radiation therapy.

The reduction in LVEF followed by persistent LVEF decline or lack of recovery (despite optimal heart failure treatment) is associated with a subsequent risk of major adverse cardiovascular events [15]. Despite this relatively high incidence, asymptomatic CTRCD is much more common during cancer therapy than symptomatic heart failure. In our study, only one patient (4%) developed symptomatic heart failure during follow-up. She received trastuzumab plus pertuzumab treatment, and the main symptoms were fatigue and dyspnoea. The incidence rate of symptomatic cardiac dysfunction in our study (4%) is similar to results previously reported (2.8–3.2%) [12, 16].

Patients with asymptomatic or subclinical cardiac dysfunction will still have cardiac functional reserve and therefore tolerate less additional injury before their symptoms of cardiotoxicity become clinically apparent [17]. A previous meta-analysis [18] showed a protective effect of beta-blockers, angiotensin receptor blockers, and angiotensin-converting enzyme inhibitors on LVEF during BC treatments compared with placebo. The overall small mean differences in the LVEF over the follow-up time could be calculated as an absolute LVEF difference of up to 2.4% between intervention and placebo [18]. In addition, cardioprotective interventions were associated with fewer interruptions of trastuzumab therapy than placebo [19]. Thus, the identification and treatment of asymptomatic CTRCD is important. Earlier changes in cardiac MRI parameters compared with baseline are prognostic for subsequent CTRCD [5, 6], and the first three months of trastuzumab therapy is the most critical period for the development of cardiotoxicity [6]. Therefore, we conducted cardiotoxicity surveillance after 3 months of trastuzumab and/or pertuzumab exposure in this study and analysed the change in cardiac MRI parameters.

In our study, the cine and strain analysis showed systolic functional impairment in both the dual anti-HER2 group and the trastuzumab group. The absolute change in LV end-systolic volume was a larger contributor to the change in LVEF than LV end-diastolic volume in both the dual anti-HER2 group and the trastuzumab group, in line with a previous study [6]. No significant difference in the LV end-diastolic volume index was found between baseline and follow-up MRI examinations. This was inconsistent with data from a previous study conducted by Gong et al. [20] and Song et al. [21]. They observed an increased LV end-diastolic volume at follow-up. This disparity could be attributed to different treatment regimens. Nearly half of the patients in their studies received anthracycline-based chemotherapy. Diastolic dysfunction is more common after treatment with anthracycline [22, 23]. Our results were generally consistent with other studies by Houbois et al. [6] and Jordan et al. [24]. Jordan et al. [24] found that LV dysfunction occurs early after cardiotoxic chemotherapy. The observed LVEF declines were driven by increases in LV end-systolic volume rather than declines in end-diastolic volume that may occur in patients with cancer who are in poor health and can exhibit intravascular volume depletion. Houbois et al. [6] also found that absolute change in LV end-systolic volume was a larger contributor to change in LVEF than LV end-diastolic volume.

GLS is considered a marker of early LV subclinical dysfunction [11]. It can accurately predict a later decrease in LVEF [25]. In our study, absolute GLS decreased significantly in the dual anti-HER2 group but not in the trastuzumab group. Pertuzumab might increase cardiotoxicity when used in combination with trastuzumab. This is supported by the recent finding that pertuzumab was associated with an approximately twofold increased risk of heart failure [26].

Native T1 relaxation times can identify myocardial fibrosis and T2 relaxation times can identify myocardial oedema. A few previous studies have investigated the anti-HER2 target therapeutic effect on myocardial T1 and T2 relaxation times. However, most of these studies had only one anti-HER2 target therapy (anthracycline), and some of the participants were previously treated with anthracycline, which may prolong T1 and T2 relaxation times. Jordan et al. showed that T1-weighted signal intensity was increased in participants previously treated with anthracycline chemotherapy, which could lead to acute, subacute, chronic myocellular injury [24]. An experimental study in a pig model of anthracycline-induced cardiotoxicity found a significant increase in T2 from week 6 and a significant increase in T1 from week 12 onwards [27]. In our study, we also found an increase in T1 and T2 relaxation times in 24 patients. However, the increases in T1 and T2 relaxation times were not significant in the dual anti-HER2 group or trastuzumab group. The lack of significance might be due to the small sample size. These findings are in concordance with a recent study by Altaha et al. [10], who noted that at the individual patient level, the mean temporal changes in cardiac MRI-measured T1 and T2 overlapped with the variability in healthy participants. Therefore, although the changes in quantitative cardiac MRI parameters may be valuable in identifying myocardial injury during cancer therapy in populations, they may be limited in individual patients or studies with small sample sizes.

Cardiac MRI facilitates early diagnosis of cancer treatment-related cardiotoxicity, but it is not a routine assessment in patients undergoing cancer treatment. Although standard cardiotoxicity surveillance every 3 months is recommended during therapy [28], we and others have noticed that in daily practice, follow-up was not always very strictly performed, especially by using cardiac MRI [29]. Previous studies have proposed different approaches to cardiotoxicity detection, trying to assess data that are surrogate from cardiac MRI. Cardiac computed tomography (CT) is a rapidly evolving technology for providing information on cardiac structures, myocardial deformation, extracellular volume and coronary vasoreactivity [30]. A previous study showed good reproducibility of CT strain measurements and detected a good correlation between CT GLS and MRI GLS [31]. Coronary CT angiography may be utilised in patients with a prior history of chemotherapy and new onset LV dysfunction to exclude underlying coronary artery disease, allowing implementation of cardiovascular risk reduction strategies prior to initiation of anthracycline based therapy. Coronary CT angiography can reliably assess epicardial coronary diameter in response to pharmacological stressors, providing a noninvasive functional index of coronary vasoreactivity. An early impairment in microvascular responses in anthracycline-induced cardiotoxicity was detected by Feher et al. [32].

Several limitations of our study need to be addressed. First, our study included 24 participants and 48 cardiac MRI scans. Therefore, the statistical power was limited due to the small sample size in this preliminary study. Moreover, diastolic function and right ventricular function were not analysed. However, Gong et al. [20] found no significant temporal change in diastolic strain rate measurements up to 18 months. Monitoring LV systolic function alone may not be sufficient to detect early right ventricular injury. Further studies are warranted. Furthermore, we did not analyse cardiac biomarkers. However, a previous study showed that cardiac biomarkers did not appear to have convincing utility for cardiotoxicity risk assessment [33]. In addition, in this preliminary study, we did not have long-term follow-up data or complete cancer therapy; hence, the long-term cardiac side effects of anti-HER2 therapy could not be assessed. However, the aim of our study was to identify early changes in LV function and tissue characterisation using cardiac MRI, so the short follow-up time can reflect the early and subtle cardiotoxicity effect. Finally, it is important to determine whether early subclinical changes in cardiac MRI parameters can predict further overt cardiac dysfunction. This is currently under study.

The aim of this preliminary study was to evaluate and compare early changes in LV function and tissue characterisation using cardiac MRI in BC patients treated with nonanthracycline-based anti-HER2 target therapy. Systolic dysfunction in this group of patients was quite frequent but had a modest clinical impact. The change of myocardial strain was more marked in patients treated with dual anti-HER2 therapy. Cardiotoxicity surveillance through MRI is an interesting option particularly in patients treated with dual anti-HER2 therapy, allowing clinicians to incorporate cardioprotective therapy and decrease the risk of therapy interruptions. Studies with larger sample sizes are warranted.

## Data Availability

The datasets used and/or analysed during the current study are available from the corresponding author on reasonable request.

## Abbreviations

BC: Breast cancer
CTRCD: Cancer therapy-related cardiovascular dysfunction
GCS: Global circumferential strain
GLS: Global longitudinal strain
GRS: Global radial strain
HER2: Human epidermal growth factor receptor 2
IQR: Interquartile range
LV: Left ventricular
LVEF: LV ejection fraction
MRI: Magnetic resonance imaging

## References

[1] Baselga J, Cortés J, Kim S-B (2012). Pertuzumab plus trastuzumab plus docetaxel for metastatic breast cancer. N Engl J Med.

[2] Howie LJ, Scher NS, Amiri-Kordestani L (2019). FDA approval summary: pertuzumab for adjuvant treatment of HER2-positive early breast cancer. Clin Cancer Res.

[3] Herrmann J (2020). Adverse cardiac effects of cancer therapies: cardiotoxicity and arrhythmia. Nat Rev Cardiol.

[4] Lyon AR, Lopez-Fernandez T, Couch LS (2022). 2022 ESC Guidelines on cardio-oncology developed in collaboration with the European Hematology Association (EHA), the European Society for Therapeutic Radiology and Oncology (ESTRO) and the International Cardio-Oncology Society (IC-OS). Eur Heart J Cardiovasc Imaging.

[5] Banke A, Schou M, Ewertz M (2021). Immediate evaluation of global longitudinal strain at initiation of trastuzumab treatment in breast cancer patients. Echocardiography.

[6] Houbois CP, Nolan M, Somerset E (2021). Serial cardiovascular magnetic resonance strain measurements to identify cardiotoxicity in breast cancer: comparison with echocardiography. JACC Cardiovasc Imaging.

[7] Greenlee H, Iribarren C, Rana JS (2022). Risk of cardiovascular disease in women with and without breast cancer: the pathways heart study. J Clin Oncol.

[8] Seidman A, Hudis C, Pierri MK (2002). Cardiac dysfunction in the trastuzumab clinical trials experience. J Clin Oncol.

[9] Plana JC, Galderisi M, Barac A (2014). Expert consensus for multimodality imaging evaluation of adult patients during and after cancer therapy: a report from the American Society of Echocardiography and the European Association of Cardiovascular Imaging. Eur Heart J Cardiovasc Imaging.

[10] Altaha MA, Nolan M, Marwick TH (2020). Can quantitative CMR tissue characterization adequately identify cardiotoxicity during chemotherapy?. JACC Cardiovasc Imaging.

[11] Zamorano JL, Lancellotti P, Rodriguez Munoz D (2016). 2016 ESC Position Paper on cancer treatments and cardiovascular toxicity developed under the auspices of the ESC Committee for Practice Guidelines: The Task Force for cancer treatments and cardiovascular toxicity of the European Society of Cardiology (ESC). Eur Heart J.

[12] Tarantini L, Cioffi G, Gori S (2012). Trastuzumab adjuvant chemotherapy and cardiotoxicity in real-world women with breast cancer. J Card Fail.

[13] Bouwer NI, Liesting C, Kofflard MJM (2021). 2D-echocardiography vs cardiac MRI strain: a prospective cohort study in patients with HER2-positive breast cancer undergoing trastuzumab. Cardiovasc Ultrasound.

[14] Esmaeilzadeh M, Urzua Fresno CM, Somerset E (2022). A combined echocardiography approach for the diagnosis of cancer therapy-related cardiac dysfunction in women with early-stage breast cancer. JAMA Cardiol.

[15] Herrmann J, Lenihan D, Armenian S (2022). Defining cardiovascular toxicities of cancer therapies: an International Cardio-Oncology Society (IC-OS) consensus statement. Eur Heart J.

[16] Lidbrink E, Chmielowska E, Otremba B (2019). A real-world study of cardiac events in > 3700 patients with HER2-positive early breast cancer treated with trastuzumab: final analysis of the OHERA study. Breast Cancer Res Treat.

[17] Omland T, Heck SL, Gulati G (2022). The role of cardioprotection in cancer therapy cardiotoxicity: JACC: CardioOncology state-of-the-art review. JACC CardioOncol.

[18] Lewinter C, Nielsen TH, Edfors LR (2022). A systematic review and meta-analysis of beta-blockers and renin–angiotensin system inhibitors for preventing left ventricular dysfunction due to anthracyclines or trastuzumab in patients with breast cancer. Eur Heart J.

[19] Pituskin E, Mackey JR, Koshman S (2017). Multidisciplinary approach to novel therapies in cardio-oncology research (MANTICORE 101-Breast): a randomized trial for the prevention of trastuzumab-associated cardiotoxicity. J Clin Oncol.

[20] Gong IY, Ong G, Brezden-Masley C (2019). Early diastolic strain rate measurements by cardiac MRI in breast cancer patients treated with trastuzumab: a longitudinal study. Int J Cardiovasc Imaging.

[21] Song L, Brezden-Masley C, Ramanan V (2019). Serial measurements of left ventricular systolic and diastolic function by cardiac magnetic resonance imaging in patients with early stage breast cancer on trastuzumab. Am J Cardiol.

[22] Serrano JM, Gonzalez I, Del Castillo S (2015). Diastolic dysfunction following anthracycline-based chemotherapy in breast cancer patients: incidence and predictors. Oncologist.

[23] Minotti G, Reggiardo G, Camilli M, Salvatorelli E, Menna P (2022). From cardiac anthracycline accumulation to real-life risk for early diastolic dysfunction: a translational approach. JACC CardioOncol.

[24] Jordan JH, D'Agostino RB, Hamilton CA (2014). Longitudinal assessment of concurrent changes in left ventricular ejection fraction and left ventricular myocardial tissue characteristics after administration of cardiotoxic chemotherapies using T1-weighted and T2-weighted cardiovascular magnetic resonance. Circ Cardiovasc Imaging.

[25] Negishi K, Negishi T, Hare JL, Haluska BA, Plana JC, Marwick TH (2013). Independent and incremental value of deformation indices for prediction of trastuzumab-induced cardiotoxicity. J Am Soc Echocardiogr.

[26] Alhussein MM, Mokbel A, Cosman T (2021). Pertuzumab cardiotoxicity in patients with HER2-positive cancer: a systematic review and meta-analysis. CJC Open.

[27] Galán-Arriola C, Lobo M, Vílchez-Tschischke JP (2019). Serial magnetic resonance imaging to identify early stages of anthracycline-induced cardiotoxicity. J Am Coll Cardiol.

[28] Celutkiene J, Pudil R, Lopez-Fernandez T (2020). Role of cardiovascular imaging in cancer patients receiving cardiotoxic therapies: a position statement on behalf of the Heart Failure Association (HFA), the European Association of Cardiovascular Imaging (EACVI) and the Cardio-Oncology Council of the European Society of Cardiology (ESC). Eur J Heart Fail.

[29] Seferina SC, de Boer M, Derksen MW (2016). Cardiotoxicity and cardiac monitoring during adjuvant trastuzumab in daily dutch practice: a study of the southeast netherlands breast cancer consortium. Oncologist.

[30] Feher A, Baldassarre LA, Sinusas AJ (2022) Novel cardiac computed tomography methods for the assessment of anthracycline induced cardiotoxicity. Front Cardiovasc Med 9:875150. 10.3389/fcvm.2022.875150 10.3389/fcvm.2022.875150 PMC9094702 35571206

[31] Wang R, Fang Z, Wang H et al (2021) Quantitative analysis of three-dimensional left ventricular global strain using coronary computed tomography angiography in patients with heart failure: comparison with 3T cardiac MR. Eur J Radiol 135:109485. 10.1016/j.ejrad.2020.109485 10.1016/j.ejrad.2020.109485 33401113

[32] Feher A, Boutagy NE, Stendahl JC (2020). Computed tomographic angiography assessment of epicardial coronary vasoreactivity for early detection of doxorubicin-induced cardiotoxicity. JACC CardioOncol.

[33] Posch F, Niedrist T, Glantschnig T et al (2022) Left ventricular ejection fraction and cardiac biomarkers for dynamic prediction of cardiotoxicity in early breast cancer. Front Cardiovasc Med 9:933428. 10.3389/fcvm.2022.933428 10.3389/fcvm.2022.933428 PMC9424929 36051281

